# Extracellular vesicle–mediated immunomodulation and targeted delivery: breakthroughs and challenges in rheumatoid arthritis therapy

**DOI:** 10.3389/fimmu.2026.1813097

**Published:** 2026-07-08

**Authors:** Bo Yang, Heguo Yan, Ye Zhou, Jiajie Li, Juncheng Liu, Changxing Huang, Zhaohu Xie, Deyong Zheng, Zhaofu Li

**Affiliations:** 1 Zhaotong Hospital of Traditional Chinese Medicine, Zhaotong, Yunnan, China; 2 Yunnan University of Chinese Medicine, Kunming, Yunnan, China

**Keywords:** extracellular vesicles, immune microenvironment, immunoregulation, mesenchymal stem cells, nanocarriers, rheumatoid arthritis, targeted delivery

## Abstract

Rheumatoid arthritis (RA) is a chronic autoimmune disorder characterized by persistent synovitis, immune dysregulation, and progressive joint destruction. Its pathogenesis is multifactorial and primarily involves disruption of immune homeostasis, accompanied by sustained activation of the local inflammatory microenvironment within the joints. Adverse effects, therapeutic resistance, and suboptimal efficacy limit the effectiveness of current clinical interventions. Extracellular vesicles (EVs), including exosomes, microvesicles, and apoptotic bodies, are natural vesicular carriers with intrinsic biocompatibility, low immunogenicity, and efficient intercellular communication capacity. By delivering diverse bioactive molecules, EVs modulate immune cell activation and functional equilibrium and enable targeted delivery of therapeutic agents to RA lesion sites via surface marker modification or functional molecule engineering. This strategy represents an emerging direction in rheumatology and immunology research. This review systematically summarizes recent progress in the immunoregulatory mechanisms and targeted delivery applications of EVs in RA, examines the technical limitations and major challenges associated with clinical translation, and outlines a theoretical foundation and research perspectives for advancing precision and personalized therapeutic strategies for RA.

## 1 Introduction

Rheumatoid arthritis (RA) is a chronic autoimmune disorder characterized by symmetrical polyarticular inflammation (1). The global prevalence is approximately 1%, whereas in China it ranges from 0.2% to 0.4%. The disease predominantly affects women between 35 and 50 years of age, with a male-to-female ratio of approximately 1:4 (2, 3). The principal pathological features involve immune dysfunction that induces synovial hyperplasia and choroid plexus formation, leading to progressive cartilage erosion and bone destruction. In advanced stages, RA leads to joint deformity and severe functional impairment. The disease may also involve multiple organ systems, including the cardiovascular, pulmonary, and hematological systems, which further compromise patient health. These complications substantially reduce quality of life and impose a significant socioeconomic burden (4, 5). Current therapeutic strategies mainly include disease-modifying antirheumatic drugs (DMARDs), glucocorticoids, and biologics. Conventional agents are associated with systemic immunosuppression, gastrointestinal injury, and osteoporosis. Although biologics selectively target defined inflammatory pathways, their clinical application is limited by high cost, increased risk of infection, insufficient therapeutic response in certain patients, and drug resistance. A definitive curative therapy remains unavailable (6–8).

Extracellular vesicles (EVs), recognized as mediators of intercellular communication and natural nanocarriers, show high biocompatibility, low immunogenicity, and the capacity to cross biological barriers. EVs transport diverse bioactive molecules, including proteins, lipids, and nucleic acids. By modulating immune cell activity, inflammatory responses, and target-cell function, EVs contribute to RA pathogenesis and immune homeostasis (9, 10). With the expansion of research in recent years, the regulatory functions of EVs in RA have been increasingly elucidated, demonstrating considerable potential for immunomodulatory therapy and targeted drug delivery. Although numerous studies have investigated the association between EVs and RA, critical research gaps remain, including fragmented mechanistic interpretation, unsystematic subtype classification, delayed integration of cutting-edge technologies, and ambiguous strategies for clinical translation. These limitations hinder a comprehensive and accurate overview of the overall research landscape and developmental trends of EVs in RA. Accordingly, we retrieved literature from PubMed, Scopus, and Web of Science to summarize the latest 5-year advances regarding EV-mediated immunomodulatory mechanisms and targeted delivery systems for RA. In this work, we refined the classification system of EV subtypes, clarified distinct immunoregulatory effects and dual pathological functions of cell-specific EVs in RA, and elaborated progress in innovative technologies such as nanoengineering modification and targeted drug loading. Furthermore, focusing on core bottlenecks restricting EV clinical translation, we analyzed prevailing obstacles from the perspectives of production quality control, *in-vivo* biosafety and regulatory supervision. A complete research framework covering mechanism, technology, and translational application was established, alongside a stepwise translational strategy. We also discussed prospective breakthroughs and pending challenges of EV-based RA therapies, providing substantial theoretical references and research clues for the development and clinical transformation of precise and individualized therapeutic regimens against RA.

## 2 Biological characteristics of EVs and their role in immune regulation

### 2.1 Classification and biological features of EVs

EVs constitute a highly heterogeneous population of nanoparticles that can be classified into three principal subgroups based on their biogenesis pathways, size, and molecular characteristics (11). Exosomes, with diameters of 30–150 nm, are generated through the endosomal pathway and released following maturation within multivesicular bodies and fusion with the plasma membrane. Microvesicles measure 100–1,000 nm in diameter and are formed by direct outward budding of the plasma membrane. Apoptotic bodies are larger vesicles, 1–5 μm in diameter, produced during programmed cell death and contain organelle fragments and nuclear components (12–15). Recent investigations have also identified smaller non-membranous nanoparticles, such as exosomal particles and ultravesicles, which differ from conventional EVs in molecular composition, formation mechanisms, and biological functions (16). The biogenesis mechanisms of EV subpopulations are distinct. Exosome formation and release depend on regulatory systems, including the endosomal sorting complex required for transport (ESCRT). In yeast models, disruption of the ESCRT machinery markedly reduces exosome production and functional transfer (17). In mammalian cells, exosome secretion is closely linked to exocytosis of multivesicular bodies (18). Microvesicles, also referred to as ectosomes, originate directly from the plasma membrane without involvement of the endosomal pathway (19). EV release is further modulated by cellular physiological conditions and external stimuli, such as heat stress and physical exercise, which influence secretion efficiency and cargo composition (20, 21).

EVs transport and preserve diverse bioactive molecules derived from their parental cells, forming the basis of their functional properties (22, 23). These include transmembrane marker proteins such as CD9, CD63, and CD81; cytoplasmic proteins indicative of cellular origin (24); nucleic acids, including mRNA and miRNA, with miRNA serving as potential biomarkers for cancers and cardiovascular diseases; and signaling lipids and metabolites (25, 26). EVs are internalized by recipient cells through membrane fusion, endocytosis, or receptor–ligand interactions, enabling delivery of functional cargo and mediating both local and long-distance intercellular communication. They participate extensively in physiological and pathological processes, including maintenance of tissue homeostasis, immune regulation, developmental processes, metabolic regulation, and antigen presentation. This communication mechanism is evolutionarily conserved from yeast to parasites, suggesting an origin in early life forms (19, 27).

EVs are implicated in multiple disease contexts. They promote tumor progression and metastasis and function as liquid biopsy biomarkers (28–30). They are also implicated in cardiovascular diseases (14) and neurodegenerative disorders (26). In autoimmune inflammation and infectious diseases, they exert bidirectional pro-inflammatory and anti-inflammatory regulatory effects (31–33). These properties establish a theoretical framework and translational potential for early diagnosis, targeted therapeutic strategies, and the development of natural drug delivery systems.

### 2.2 Role of EVs in immune regulation

As central mediators of immune communication, EVs function as intercellular messengers. The immune-active molecules they transport, including MHC class I/II molecules, cytokines, and self-antigens, directly modulate immune cell activity. EVs contribute not only to local immune regulation but also to the immune status of distant tissues. For example, in lung transplantation, EVs released by donor cells can carry lung self-antigens and participate in immune processes associated with acute rejection and chronic graft dysfunction (34). In cancer, tumor-derived EVs (TDEVs) can establish a “pre-metastatic niche” that facilitates metastasis (35). EVs regulate both innate and adaptive immunity, enabling multidimensional modulation that can activate or suppress immune responses and support antigen presentation (36). These effects are primarily mediated through key signaling pathways. In inflammatory signaling, TDEVs modulate NLRP3 inflammasome activity, affecting inflammatory responses and promoting immune evasion within the tumor microenvironment. They also transmit signals through the NF-κB pathway, thus enhancing inflammation and contributing to immune suppression (37).

For immunosuppressive regulation, TDEVs inhibit immune cell effector functions and alter immune cell recruitment and polarization via pathways such as JAK-STAT and PI3K/Akt/mTOR, hence facilitating tumor progression and immune escape (37). This process converts “hot tumors” into “cold tumors,” creating an immunosuppressive microenvironment (38). Regarding immune cell fate, EVs derived from apoptotic cells (ApoEVs) convey diverse bioactive molecules that modulate inflammatory and immune programs and cell fate (39). In phagocytic processes, EVs are critical for apoptotic cell clearance, influencing macrophage polarization, calreticulin (CRT) expression, and TAM receptor activity (40).

EVs also exert critical functions in specific physiological and pathological contexts. During pregnancy, placental and embryonic EVs participate in endocrine regulation and maternal–fetal immune communication, which are essential for maintaining normal gestation. Alterations in their composition are associated with pregnancy complications such as preeclampsia and gestational diabetes (27, 41). Placental EVs transport corticotropin-releasing hormone (CRH) mRNA into maternal circulation, providing potential for pregnancy monitoring (42). In inflammatory skin disorders, including psoriasis, atopic dermatitis, and systemic lupus erythematosus, EVs induce cytokine release, modulate immune cell activity, and trigger inflammatory and immune responses, hence contributing to disease progression (43). In infection immunity, EVs secreted by pathogens including Giardia lamblia with distinct lipidome profiles may mediate host-parasite crosstalk. It has been verified that these EVs regulate the innate immunity of host cells through the TLR2 and NLRP3 inflammasome signaling pathways and alleviate inflammatory infection (44).

EVs show considerable promise in biomedical diagnostics and therapeutic applications owing to their immunomodulatory properties. Exosomes, in particular, display remarkable stability and accurately reflect the physiological state of their parent cells, supporting their use in liquid biopsy approaches. Certain EV-associated proteins and miRNAs have been identified as diagnostic and prognostic markers in cancers such as melanoma, glioma, and pancreatic cancer (45). In the context of organ transplantation, circulating EV-derived molecules, including miRNAs, are being explored as non-invasive indicators of graft rejection (46).

As therapeutic agents and delivery vehicles, EVs possess inherent biocompatibility and the ability to cross biological barriers (47, 48). Mesenchymal stem cell–derived EVs (MSC-EVs) demonstrate significant therapeutic potential; for instance, in lung transplantation, they can mitigate primary graft dysfunction by reducing inflammation and alleviating ischemia–reperfusion injury (34). They also facilitate tissue and organ repair, attenuate inflammation and apoptosis, and modulate immune responses (49). In neuroregeneration, stem cell–derived EVs contribute to neural repair and function as carriers for bioactive molecules or drugs (50). Furthermore, engineered EVs are actively being explored for targeted therapy and immunomodulatory applications (43).

### 2.3 Isolation, purification, and characterization of EVs

The isolation, purification, and characterization of EVs are fundamental for advancing EV research and applications. Due to their small size, low abundance, and tendency to co-isolate with impurities in bodily fluids, such as proteins and lipoproteins, obtaining high-purity EVs and establishing standardized characterization systems remain major challenges.

Separation and purification methods primarily exploit the physical properties of EVs. Gradient-speed ultracentrifugation, a classical approach, enables enrichment of distinct EV subpopulations by applying defined rotational-speed gradients. Its limitations include the time-consuming nature of the process; the risk that EVs may be damaged by mechanical forces during centrifugation, which can impair their biological functions; and the fact that the purified EVs may not be sufficiently pure, potentially containing small amounts of impurities such as lipoproteins (51). Size-exclusion chromatography (SEC) effectively removes soluble protein contaminants and is widely used to isolate highly purified small EVs, such as exosomes; however, this method has limited sample loading capacity, results in severe sample dilution after elution, and has low throughput, making it difficult to scale up for large-scale preparation. Furthermore, samples with high levels of impurities are prone to clogging the packing material, leading to reduced separation efficiency (52–54). Sequential ultrafiltration with regenerated cellulose membranes can be employed for small EV preparation, whereas ultrafiltration preconcentration combined with SEC is suitable for purifying complex samples such as plant juices. However, the ultrafiltration process is prone to causing the adsorption and aggregation of extracellular vesicles and membrane degradation, cellulose membranes are susceptible to protein contamination, and the combined process is cumbersome to operate and lacks versatility (55). For large-scale production, tangential flow filtration (TFF) coupled with anion exchange chromatography (AEX) offers a scalable strategy comparable to traditional ultracentrifugation. However, this system is significantly affected by buffer pH and ion strength, and separation based solely on charge differences makes it difficult to completely eliminate impurity vesicles with similar charges. Additionally, this approach suffers from issues such as vesicle compression and rupture, insufficient removal of small-molecule proteins, and high costs for packing materials and equipment (56). Bioengineered adsorption filters can also selectively remove pathogenic microvesicles (~200 nm) from blood; however, because they have a single sorting particle size, they simultaneously retain some larger-sized small EVs, resulting in product loss. They are suitable only for blood samples, have a narrow range of applicable matrices, are prone to rapid saturation of the packing material, and have a limited single-treatment capacity (57).

EV identification follows the International Society for Extracellular Vesicles (ISEV) guidelines, emphasizing morphology, size distribution, and marker proteins. Morphological characterization was performed using transmission electron microscopy (TEM) and scanning electron microscopy (SEM). TEM is the gold standard for morphological characterization, as it clearly reveals the morphology, size, and bilayer lipid membrane structure of EVs, while SEM is used to observe surface morphology and distribution. Both methods offer the advantages of high resolution and the ability to visually demonstrate the characteristics of EVs; however, they are limited by complex procedures, time-consuming analysis, sample preparation that can easily damage EVs, and the inability to perform quantitative analysis (58). Nanoparticle tracking analysis (NTA) and dynamic light scattering (DLS) are commonly used for particle size distribution analysis. NTA can track the movement trajectories of vesicles and simultaneously and accurately detect the particle size distribution and concentration of EVs. DLS relies on laser scattering to analyze particle size but is susceptible to interference from sample impurities, resulting in lower detection accuracy. For the identification of signature proteins, Western blot, flow cytometry (FCM), and enzyme-linked immunosorbent assay (ELISA) can be used. All three methods can detect EV-specific proteins such as CD9, CD63, CD81, HSP70, and other EV-specific proteins. Western blot is a commonly used method for protein identification, while flow cytometry allows for rapid detection and quantification; however, due to the minute size of EVs, specialized equipment is required. ELISA is simple to perform and highly sensitive, capable of quantifying proteins; its drawback is relatively poor specificity, making it susceptible to interference from sample contaminants (52, 53). Current EV research emphasizes integrating multiple techniques and developing highly selective, scalable methods that preserve intrinsic EV properties. This approach increases understanding of EV heterogeneity and biological function, providing technical support for translating fundamental EV research into clinical applications (**Table 1**) (59).

**Table 1:** Advantages and limitations of mainstream isolation, purification and identification technologies for EVs.

Technology category	Technology name	Applicable EVs subtypes	Advantages	Limitations	References
Isolation and purification	Differential ultracentrifugation	Full-spectrum EVs subtypes (exosomes, microvesicles, apoptotic bodies)	Classic and universal method, wide sample applicability, capable of separating EVs subpopulations by particle size	Time-consuming, relatively low purity, prone to co-precipitation with lipoprotein impurities	(51)
Isolation and purification	Size exclusion chromatography (SEC)	Small EVs (exosomes)	high removal efficiency of soluble protein contaminants, well-preserved EVs structural integrity	limited sample loading capacity, difficult for industrial large-scale preparation	(52, 53)
Isolation and purification	Tangential flow filtration (TFF) coupled with anion exchange chromatography (AEX)	Large-scale industrial production of EVs	scalable for industrialization, high production capacity, purification effect comparable to ultracentrifugation	High equipment cost, requiring supporting process optimization	(56)
Identification technology	Combined identification of transmission electron microscopy (TEM), nanoparticle tracking analysis (NTA) and Western blot	Full-spectrum EVs subtypes	gold standard recommended by the international society for extracellular vesicles (ISEV), realizing trinity identification of morphology, particle size and biomarkers	High detection cost, cumbersome operation, not suitable for high-throughput detection	(52, 58)

## 3 EV-mediated immunoregulatory mechanisms in RA

The hallmark pathological features of RA include persistent synovial inflammation and progressive joint tissue damage driven by immune dysregulation. This dysfunction is characterized by abnormal activation of innate immune cells, including macrophages, neutrophils, and dendritic cells (DCs), as well as adaptive immune cells, such as T cells and B cells (60, 61). It also involves imbalances in pro-inflammatory and anti-inflammatory cytokine networks and the aberrant proliferation and invasive behavior of fibroblast-like synoviocytes (FLSs) (62, 63). In the pathological microenvironment of RA, multiple cell types including immune cells, FLSs, platelets, and mesenchymal stem cells can secrete extracellular vesicles (EVs). EVs derived from distinct cell sources carry specific bioactive molecules and exert divergent regulatory functions: EVs secreted by immune cells primarily mediate the amplification of inflammatory cascades and the modulation of immune dysregulation; FLS-derived EVs dominate aberrant synovial proliferation and persistent local joint inflammation; platelet-derived EVs are involved in inflammatory activation, coagulation disorders and articular injury; mesenchymal stem cell–derived EVs feature core functions of broad-spectrum anti-inflammation, immune remodeling and tissue repair. Interconnected with one another via synergistic or antagonistic interactions, all subtypes of EVs coordinately govern the pathological progression of RA. As central mediators of intercellular communication, EVs can modulate these pathological processes via the bioactive molecules they carry, including proteins and nucleic acids. Through these mechanisms, EVs contribute to the restoration of immune homeostasis in RA, attenuate synovial inflammation, and mitigate joint tissue damage (64).

### 3.1 Regulation of innate immune cells

**Figure 1:**
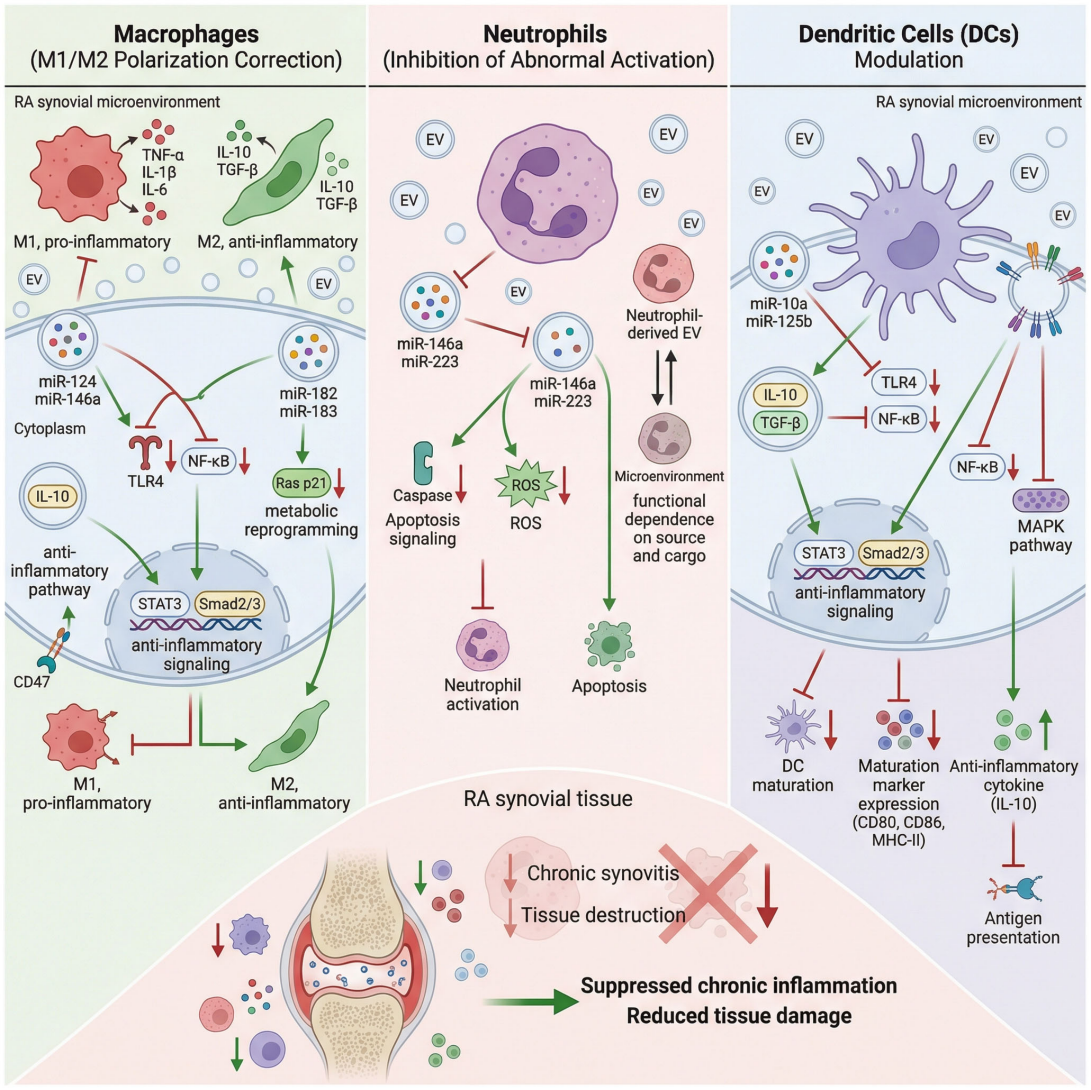
Regulatory mechanisms of EVs on innate immune cells in RA. EVs modulate innate immune cells in RA through multiple targeted pathways. Macrophages: EVs correct M1/M2 polarization imbalance via miRNAs (e.g., miR-124, miR-146a, miR-182/183), cytokines (e.g., IL-10), and membrane receptors (e.g., CD47). These actions suppress pro-inflammatory M1 polarization, promote anti-inflammatory M2 polarization, and restore balance. Neutrophils: EVs carry miR-146a and miR-223 to regulate inflammatory genes, activate apoptotic pathways, and provide antioxidant effects. Their effects depend on the microenvironment and cargo, suppressing overactivation and promoting apoptosis. DCs: EVs deliver miR-10a and miR-125b, anti-inflammatory cytokines (IL-10, TGF-β), and engage receptors to inhibit NF-κB and MAPK signaling. This suppresses DC maturation, reduces pro-inflammatory cytokine secretion, increases anti-inflammatory mediators, and limits antigen presentation. EVs reduce persistent synovial inflammation and protect joint tissue in RA.

The innate immune system constitutes the first line of defense against pathogens and foreign antigens and plays a key role in initiating synovial inflammation in RA. In this disease, innate immune cells, including macrophages, neutrophils, and DCs, become aberrantly activated, producing excessive pro-inflammatory cytokines that sustain inflammation and exacerbate joint tissue damage (**Table 2**). EVs regulate the activity of these innate immune cells through multiple mechanisms, suppressing their activation and promoting their polarization toward anti-inflammatory phenotypes, leading to the mitigation of synovial inflammation (**Figure 1**).

**Table 2:** Summary of contents, targets, and biological effects of EVs from different sources in rheumatoid arthritis.

EVs source type	Core bioactive cargo components	Key targets/signaling pathways	Target immune cells	*In-vitro* and *in-vivo* biological effects	References
Mesenchymal stem cell–derived EVs (MSC-EVs)	miR-124, miR-146a, miR-125b, miR-34a; IL-10, TGF-β, antioxidant enzymes (SOD, catalase), membrane integrins	TLR4/NF-κB, NLRP3 inflammasome, JAK/STAT3, PI3K/Akt/mTOR, RORγt, Arg-1/CD206	Macrophages, neutrophils, dendritic cells (DCs), CD4^+^ T cells (Th17/Treg), B cells, fibroblast-like synoviocytes (FLSs)	① Induce M1-to-M2 macrophage polarization, downregulate TNF-α, IL-6, and IL-1β; ② Inhibit DC maturation, downregulate CD80/CD83/CD86/MHC-II expression;③ Restore the Th17/Treg balance, suppress Th17 differentiation, and expand Treg cells;④ Inhibit plasma cell differentiation of B cells, reduce RF and ACPA secretion;⑤ Suppress neutrophil NETs formation and reduce synovial infiltration;⑥Ameliorate joint swelling, bone and cartilage damage in the CIA model	(67, 68, 84, 98, 113, 117, 118)
Regulatory T cell–derived EVs (Treg-EVs)	IL-10, TGF-β, functional membrane ligands, regulatory miRNAs	NF-κB, STAT3, pro-inflammatory transcription pathways	Macrophages, Th17 cells, B cells	Inhibit pro-inflammatory cytokine release from macrophages and Th17 cells, promote Breg differentiation, and downregulate autoantibody synthesis	(70, 119, 120)
Neutrophil-derived EVs (nEVs)	miR-455-3p, characteristic lipidome, pro-coagulant lipids, pro-inflammatory membrane proteins	Wnt, notch, TLR signaling pathways	Synovial fibroblasts, chondrocytes, macrophages	Bidirectional regulation: inflammation-activated nEVs aggravate synovial coagulation and local inflammation; healthy donor-derived nEVs exert chondroprotective and cartilage-repairing effects, and inhibit excessive local inflammation	(81, 83, 93)
Dendritic cell–derived EVs (DC-EVs)	IL-10, costimulatory molecules (CD40L/CD86), regulatory non-coding RNAs	NF-κB, MAPK, T-cell activation pathways	T cells, macrophages	Inhibit macrophage and T-cell activation, reduce IL-6/TNF-α release, and regulate antigen presentation efficiency	(72, 121)
Probiotic-derived EVs	Lipopolysaccharide derivatives, functional small RNAs	TLR4/NF-κB	Macrophages	Block the TLR4 pathway, inhibit M1 polarization, and downregulate TNF-α/IL-6 secretion	(71, 122)
Engineered EVs (CTLA4Ig-modified/anti-CD80-conjugated/drug-loaded MSC-EVs)	Overexpressed CTLA4Ig protein, methotrexate, ROS-scavenging nanomaterials, targeting modified peptides	CD80, CTLA4-B7 pathway, ROS oxidation pathway, RORγt	Pro-inflammatory macrophages, Th cells, synovial FLSs	Specifically target and accumulate in macrophages of the inflamed joint, exert potent local anti-inflammatory effects with significantly higher efficacy than native EVs; superior amelioration of joint damage in the CIA model compared with unmodified MSC-EVs	(75, 76, 86)

#### 3.1.1 Regulation of macrophages

Macrophages are central innate immune cells driving synovial inflammation in RA. They infiltrate extensively into affected synovial tissue and display a dysregulated M1/M2 polarization balance. The M1 pro-inflammatory phenotype predominates, secreting cytokines such as TNF-α and IL-6, which promote synovial inflammation and joint destruction. In comparison, the M2 anti-inflammatory phenotype releases IL-10, TGF-β, and other factors that support tissue repair. This polarization imbalance sustains chronic synovial inflammation in RA. Therefore, restoring the M1/M2 polarization equilibrium of macrophages constitutes a key mechanism of EV-mediated immune regulation in RA (65, 66). EVs from diverse sources can significantly suppress M1 polarization and promote M2 polarization in macrophages. Among these, MSC-EVs have been most extensively investigated. They deliver miRNAs such as miR-124 and miR-146a to downregulate inflammation-associated genes, including TLR4 and NF-κB, while simultaneously upregulating M2 markers such as Arg-1 and CD206 (67). In collagen-induced arthritis (CIA) models, MSC-EVs significantly ameliorate joint inflammation and bone damage (68, 69). EVs derived from immune cells, including regulatory T cells and DCs, modulate macrophage polarization through the delivery of anti-inflammatory cytokines or co-stimulatory molecules (70). Probiotic-derived EVs similarly inhibit pro-inflammatory cytokine secretion and favor M2 polarization (71).

The molecular mechanisms underlying EV-mediated macrophage polarization are multi-pathway. First, miRNA-mediated regulation fine-tunes macrophage function, exemplified by miR-182/183 in cortical osteoblast-derived EVs, which target Ras p21 protein to induce metabolic reprogramming and pro-repair polarization (72, 73). Second, cytokine-mediated modulation activates anti-inflammatory pathways, including STAT3 and Smad2/3, via EV-delivered IL-10. Third, membrane receptor-mediated signaling, including CD47-activated pathways, further regulates macrophage responses (74).

Engineering strategies have increased the targeting efficiency and therapeutic efficacy of EVs at lesion sites. For example, anti-CD80–methotrexate–modified EVs selectively target CD80^+^ pro-inflammatory macrophages in joints (75), while MSC-EVs coated with catalytically active nanomaterials promote M1-to-M2 polarization by scavenging reactive oxygen species (76). Composite hydrogels encapsulating EVs derived from *Saussurea costus* effectively inhibit M1 polarization *in vitro* (77). The functional efficacy of EVs is also influenced by donor status; human adipose–derived stem cell EVs from obese donors show reduced anti-inflammatory activity and diminished ability to modulate macrophage polarization compared to those from lean donors (78).

EVs modulate macrophage polarization in RA through multiple sources and molecular mechanisms. Engineering modifications can further enhance their targeted therapeutic potential, whereas donor characteristics are a critical determinant of efficacy. These insights provide a theoretical basis and research direction for EV-based RA therapies, guiding donor selection and engineered optimization in future studies.

#### 3.1.2 Regulation of neutrophils

In RA, neutrophils are key innate immune cells that infiltrate the synovium, where they drive inflammation and joint tissue damage through the release of pro-inflammatory mediators, the formation of neutrophil extracellular traps (NETs), and delayed apoptosis. EVs exert multidimensional regulation over neutrophil function in RA, influencing disease onset, monitoring, and potential therapeutic intervention, therefore providing novel perspectives for RA diagnosis and treatment (79, 80).

Neutrophil-derived EVs (nEVs) show dual functions. nEVs released by activated neutrophils in the RA inflammatory microenvironment enhance procoagulant activity within the synovial fluid EV pool, associated with altered lipidomic profiles (81, 82). Compared with nEVs from healthy donors or RA patients, nEVs from healthy donors or RA patients can activate joint development and repair pathways, such as Wnt and Notch, via molecules like miR-455-3p, resulting in chondroprotective and regenerative effects. Their functional outcomes depend on the originating microenvironment and the specific cargo they carry (83). Among EVs derived from other cell types, MSC-EVs have been most extensively studied. MSC-EVs modulate the immune microenvironment by suppressing pathogenic T-cell responses, promoting macrophage M2 polarization, and inhibiting neutrophil activation and NETosis (84, 85). MSC-EVs overexpressing CTLA4Ig show enhanced therapeutic efficacy (86). Circulating EVs also serve as emerging biomarkers; changes in their concentration, size, proteomic composition, and miRNA profiles (e.g., miR-212-3p, miR-338-5p) reflect neutrophil activation states, supporting early RA diagnosis and evaluation of treatment response (87–89).

Advances in engineered EVs include strategies such as MSC-EV microneedle delivery, EV-inspired nanocarriers, and EV-coated metal-organic frameworks, which allow targeted modulation of joint immune homeostasis and reduction of neutrophil-mediated tissue damage (90–92). EVs regulate neutrophil activity by modulating inflammatory gene expression, activating apoptotic signaling pathways, and counteracting oxidative stress via cargo molecules like miR-146a and miR-223, suppressing neutrophil activation and promoting apoptosis (93, 94).

The multilevel mechanisms by which EVs regulate neutrophils in RA underscore their value for understanding disease pathology and developing therapeutic strategies, providing a foundation for future research and clinical translation.

#### 3.1.3 Regulation of DCs

DCs play a central role in RA pathogenesis and immune regulation. Evidence indicates that EV levels in the plasma of RA patients are significantly elevated and display distinct proteomic profiles compared to healthy controls. These differentially expressed proteins are enriched in inflammatory regulatory networks, suggesting that EVs may originate from immune cells, including DCs, and target these cells to propagate inflammation in RA (95). Bibliometric analyses further identify “DCs” as a high-frequency keyword in studies on EVs and RA, underscoring the critical involvement of DC-derived EVs in disease pathogenesis (96).

As professional antigen-presenting cells, DC dysfunction is a core contributor to the initiation and maintenance of RA autoimmunity. EVs can influence DC function by modulating antigen-presenting capacity and pro-inflammatory cytokine secretion through the delivery of microRNAs, long non-coding RNAs, or functional proteins, enabling bidirectional regulation of RA inflammatory responses (72). Although the precise molecular mechanisms underlying EV-mediated regulation of DCs remain incompletely defined, available evidence supports the existence of this pathway. EVs derived from M2 macrophages can selectively reprogram pro-inflammatory M1 macrophages (97), while nEVs display altered lipidomic profiles within inflamed joints and contribute to the EV composition of synovial fluid (81). These findings suggest complex interactions among EVs released by diverse immune cells within the inflammatory microenvironment, regulating the functional states of immune cells, including DCs.

From a therapeutic perspective, MSC-EVs have gained prominence due to their potential immunomodulatory effects. They suppress pathogenic T-cell responses, restore Th17/Treg balance, and promote macrophage polarization toward an anti-inflammatory phenotype (68). This immunomodulatory capacity also encompasses DCs. MSC-EVs suppress DC maturation in RA patients by downregulating the expression of CD80, CD83, CD86, and MHC-II, while decreasing the secretion of pro-inflammatory cytokines such as IL-6, IL-12, and TNF-α and enhancing the production of the anti-inflammatory cytokine IL-10. These effects diminish DC antigen-presenting capacity and their ability to drive T cell proliferation (98).

The molecular mechanisms underlying EV-mediated regulation of DC maturation in RA involve three main pathways. First, miRNAs such as miR-10a and miR-125b target DC maturation-related genes, including TLR4 and NF-κB, to suppress their expression. Second, EVs deliver anti-inflammatory cytokines, such as IL-10 and TGF-β, exerting direct modulatory effects. Third, they inhibit activation of inflammatory signaling pathways, including NF-κB and MAPK, through interactions with DC surface ligands via membrane receptors (99, 100). EVs serve as carriers of bioactive molecules that critically modulate DC function in RA. Targeting the interaction pathways between EVs and DCs represents a promising therapeutic strategy. However, the precise molecular mechanisms by which EVs regulate RA DCs remain incompletely understood, and further investigation is required to provide experimental evidence and guide immune-targeted therapeutic development in RA.

### 3.2 Regulation of adaptive immune cells

**Figure 2:**
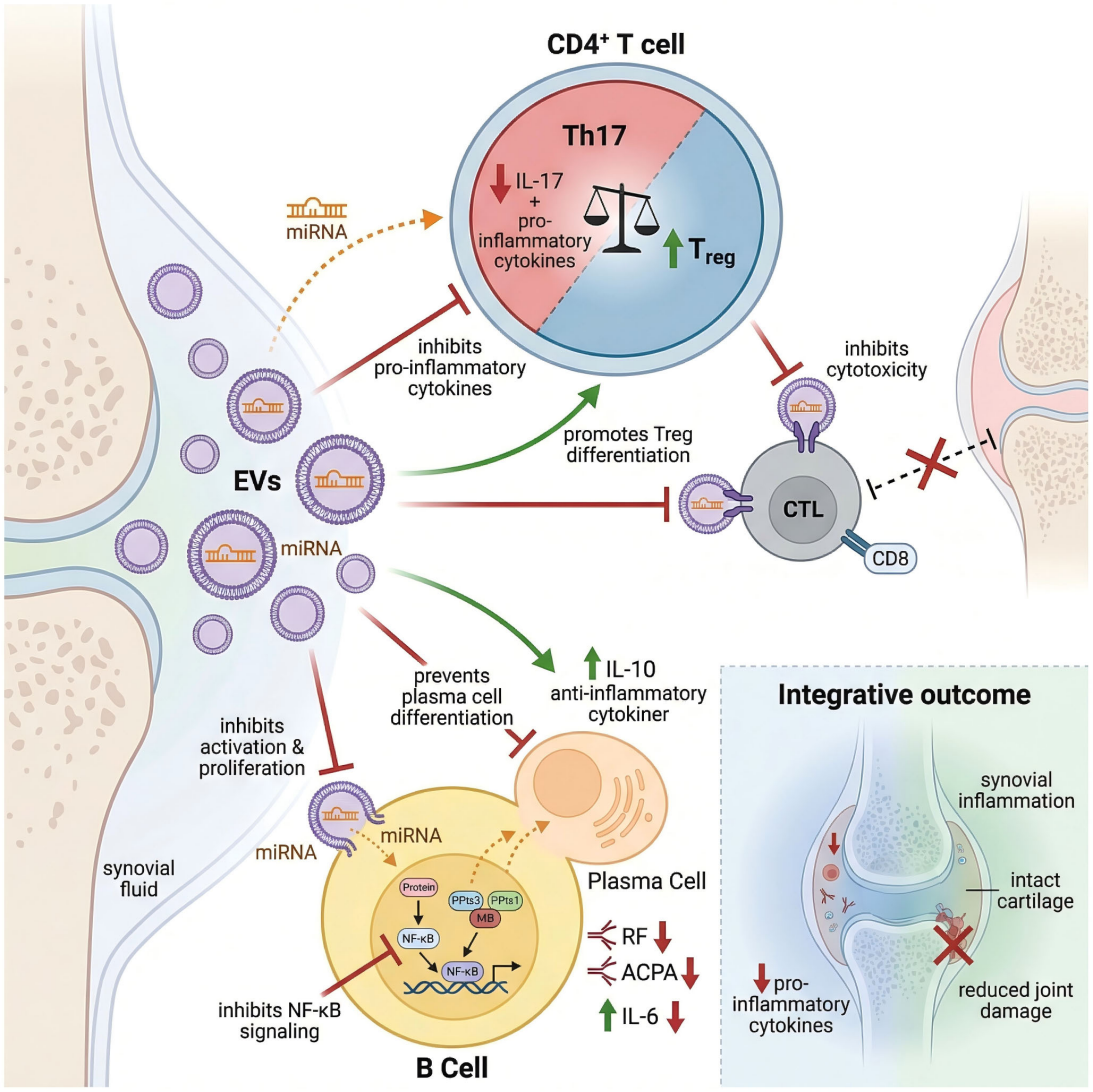
Regulatory mechanisms of EVs on adaptive immune cells in RA. EVs modulate adaptive immune cells in RA to restore immune balance and suppress excessive inflammation. T cells: EVs reduce pro-inflammatory cytokine secretion by CD4+ T cells and restore subpopulation balance through modulation of the Th17/Treg axis. They also deliver miRNAs to regulate T-cell differentiation and function, and inhibit cytotoxic T lymphocyte (CTL)–mediated joint tissue damage. B cells: EVs suppress abnormal activation, proliferation, and plasma cell differentiation. This reduces autoantibody production (e.g., RF, ACPA) and pro-inflammatory cytokine secretion (e.g., IL-6), while promoting IL-10 production. These effects are mediated by miRNAs, anti-inflammatory cytokines, and NF-κB signaling pathways. By regulating T and B cells, EVs also indirectly modulate the RA cytokine network. They suppress pro-inflammatory factors such as TNF-α and IL-6, maintain immune homeostasis, and alleviate synovial inflammation and joint damage.

Adaptive immune responses are a core component of immune dysregulation in RA, primarily involving abnormal activation of T and B cells. In RA, these cells undergo abnormal activation and proliferation, producing excessive pro-inflammatory cytokines and autoantibodies. This creates a persistent cycle of immune-mediated inflammation that accelerates joint tissue damage. EVs can modulate the function of RA adaptive immune cells through multiple pathways, restoring their equilibrium and suppressing excessive adaptive immune activation, which alleviates synovial inflammation and joint damage (**Figure 2**) (101).

#### 3.2.1 Regulation of T-cell subpopulations

As core cells in RA immune dysregulation, imbalances in T-cell subpopulations (primarily characterized by abnormal activation of Th1 and Th17 cells and reduced numbers and functional defects of Treg cells) and excessive secretion of pro-inflammatory cytokines are key pathological features that drive the persistent progression of RA inflammation. EVs can precisely regulate T-cell function through multiple pathways, providing important theoretical support for cell-based therapeutic strategies in RA (77). EVs can directly modulate CD4^+^ T-cell function and cytokine profiles, exerting immunosuppressive effects. Studies indicate that interferon-β (IFN-β)–pretreated MSC-EVs more effectively suppress the production of pro-inflammatory cytokines (including IL-4, GM-CSF, IFN-γ, IL-2, and TNF-α) by CD4^+^ T cells from RA patients compared to untreated groups, while also reducing their multifunctionality and inhibiting pathogenic T-cell responses (102). This effect has been validated in animal models, including studies of gingival-derived MSC-EVs that balance pathogenic T-cell responses and effectively alleviate arthritis symptoms (103).

Regulating the balance of the Th17/Treg pathway is a core mechanism by which EVs modulate T-cell immunity in RA and represents a key therapeutic target. MSC-EVs effectively restore the imbalanced Th17/Treg subpopulations in RA (68). Differences in Treg regulation are observed among EVs from various sources: while MSCs themselves slightly reduce Treg frequency, the EVs they secrete can restore Treg levels (102). Engineering modifications can elevate the regulatory capacity of EVs. For instance, treatment of CIA mice with MSC-EVs overexpressing CTLA4Ig (CT-EVs) significantly increased the proportion of Th2 cells and serum IL-4 levels, shifting the T-cell response toward an anti-inflammatory phenotype (86). Non-coding RNAs (especially miRNAs) carried by EVs serve as key mediators in regulating T-cell function (104). MSC-derived EVs, rich in miRNAs, can be internalized by T cells, influencing T-cell differentiation and function by regulating target-cell gene expression and signaling pathways (84). For example, miR-455-3p in nEVs exerts anti-inflammatory and chondroprotective effects by modulating the local joint immune microenvironment (including T cells) (83). Furthermore, EVs can inhibit Th1 and Th17 cell activation and proliferation, while promoting Treg cell proliferation and functional recovery via miRNAs, anti-inflammatory cytokines, and signaling pathways. They suppress CTL-mediated killing of joint tissues, therefore reducing joint damage (105). In summary, EVs profoundly regulate T-cell dysregulation in RA pathogenesis, primarily through mechanisms that include direct inhibition of pro-inflammatory cytokine secretion by CD4^+^ T cells, modulation of key subset balance (Th17/Treg), and delivery of regulatory miRNAs. These findings not only deepen our understanding of RA pathogenesis but also provide a strong theoretical basis for developing novel cell-free therapeutic strategies targeting EVs in RA, with significant translational clinical value.

#### 3.2.2 Regulation of B cells

As the core cells responsible for antibody production in adaptive immunity, B cells play a crucial role in the immune dysregulation of RA (106, 107). In RA patients, abnormally activated B cells proliferate and differentiate into plasma cells, massively secreting autoantibodies such as rheumatoid factor (RF) and anti-cyclic citrullinated peptide antibodies (ACPA). These autoantibodies bind to self-antigens to form immune complexes, which deposit in the synovium, activate the complement system, recruit infiltrating immune cells, and exacerbate synovial inflammation and joint damage (108–110). Pro-inflammatory cytokines such as TNF-α and IL-6, along with anti-inflammatory cytokines such as IL-10, are secreted by B cells and jointly participate in immune regulation and inflammatory responses (111). Therefore, suppressing abnormal B cell activation and reducing autoantibody secretion are critical steps in regulating RA immune dysregulation.

EVs can modulate RA B-cell function through multiple pathways, alleviating immune dysregulation and synovial inflammation (112). *In-vitro* studies demonstrated that 48h treatment of peripheral blood B cells from RA patients with MSC-EVs resulted in a significant reduction in B-cell proliferation (*p* < 0.05), with CD80 and CD86 positive expression decreasing by 32.1% and 28.7%, respectively. mRNA levels of plasma cell markers BLIMP-1 and IRF4 were significantly downregulated (*p* < 0.01), while concentrations of RF and ACPA in the culture supernatants decreased by 41.3% and 37.9%, respectively. IL-6 secretion was also reduced by 39.5%, and IL-10 secretion increased 2.3-fold (113). *In-vivo* experiments demonstrated that tail vein infusion of MSC-EVs into CIA mice significantly reduced serum RF and ACPA levels, decreased B-cell and plasma cell infiltration in synovial tissue, and alleviated joint swelling (114). Furthermore, Treg-EVs, DC-EVs, and SMSCs-EVs can also regulate RA B-cell function through distinct pathways. The regulatory mechanisms of EVs primarily involve three aspects: miRNA-mediated, cytokine-mediated, and signaling pathway-mediated effects. MicroRNAs such as miR-155 and miR-34a can target and suppress the expression of genes related to B-cell activation and plasma cell differentiation; anti-inflammatory cytokines like IL-10 activate B-cell anti-inflammatory signaling pathways, promoting regulatory B-cell conversion; membrane receptors carried by EVs bind to B-cell surface ligands, inhibiting inflammatory signaling pathways such as NF-κB while activating anti-inflammatory pathways (115, 116). In summary, EVs precisely regulate RA B-cell activation, proliferation, and differentiation through multiple sources and mechanisms, reducing autoantibody secretion and pro-inflammatory cytokine release while alleviating joint inflammation. This provides novel targets and strategies for the clinical treatment of RA.

### 3.3 Modulation of cytokine networks

**Figure 3:**
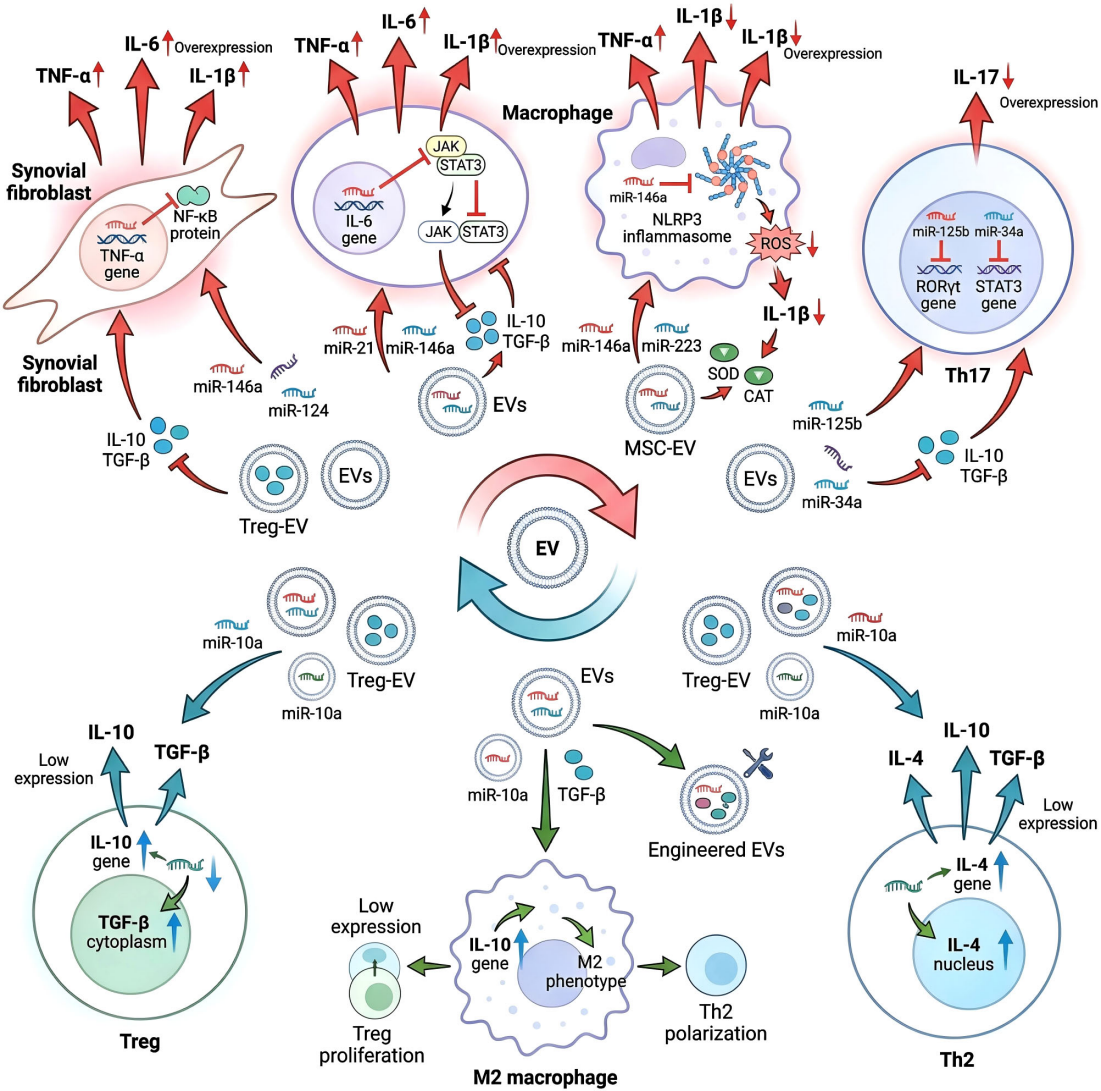
Mechanism of EVs modulating the RA cytokine network. Cytokine imbalance drives RA immune dysregulation and chronic synovial inflammation, with elevated pro-inflammatory factors such as TNF-α, IL-6, IL-1β, and IL-17, and reduced anti-inflammatory factors including IL-10, TGF-β, and IL-4. EVs restore this balance by delivering bioactive molecules such as proteins and nucleic acids, alleviating joint inflammation and tissue damage. EVs suppress TNF-α through MSC-EV–carried miR-146a and miR-124, which inhibit NF-κB, and through Treg-EV–mediated IL-10 and TGF-β signaling. IL-6 expression and secretion are reduced via miR-21, miR-146a, IL-10, TGF-β, and inhibition of the IL-6/JAK/STAT3 pathway. EVs block IL-1β maturation and release by inhibiting NLRP3 inflammasome activation with miR-146a and miR-223 and by supplying antioxidant enzymes. IL-17 secretion is suppressed by silencing RORγt and STAT3 in Th17 cells via miR-125b and miR-34a or by IL-10 and TGF-β–mediated inhibition of Th17 activation. EVs also promote IL-10, TGF-β, and IL-4 production, induce M2 macrophage and Th2 cell polarization, and expand anti-inflammatory populations such as Tregs. Engineered modifications, including gene editing or cell pretreatment, can further improve anti-inflammatory efficacy and restore immune balance in RA.

Cytokine network dysregulation is a central feature of immune imbalance and chronic synovial inflammation in RA. Lesions in RA show elevated levels of pro-inflammatory cytokines, including TNF-α, IL-6, IL-1β, and IL-17, accompanied by reduced levels of anti-inflammatory cytokines such as IL-10, TGF-β, and IL-4. This imbalance between pro- and anti-inflammatory cytokines sustains synovial inflammation and accelerates articular cartilage and bone damage (123, 124). As key mediators of intercellular communication, EVs can bi-directionally modulate the RA cytokine network via their cargo of bioactive molecules, including proteins and nucleic acids. They suppress the secretion of pro-inflammatory cytokines while enhancing anti-inflammatory cytokine expression, restoring cytokine network equilibrium, and mitigating synovial inflammation and joint tissue damage (**Figure 3**) (64, 112). This mechanism constitutes a major pathway through which EVs mediate immune regulation in RA.

#### 3.3.1 Inhibition of pro-inflammatory cytokines

Pro-inflammatory cytokines are central mediators in the initiation and progression of synovial inflammation in RA. Key cytokines, including TNF-α, IL-6, IL-1β, and IL-17, show expression levels that closely correlate with RA disease activity and are principal targets of current clinical interventions, such as biologics, including anti-TNF-α antibodies and anti-IL-6 receptor antibodies (125). EVs can significantly suppress the secretion of these pro-inflammatory cytokines via multiple pathways, resulting in the disruption of the self-amplifying cycle of inflammation.

TNF-α, the earliest identified core pro-inflammatory cytokine in RA, activates target cells, including macrophages and FLSs. It induces downstream secretion of IL-6 and IL-1β, promotes abnormal synovial cell proliferation, and elevates MMP expression, ultimately accelerating joint cartilage erosion and bone destruction, establishing its role as a key driver of RA pathogenesis (126, 127). Recent studies have shown that EVs from diverse sources effectively suppress aberrant TNF-α secretion in RA through defined mechanisms. MSC-EVs deliver miRNAs, including miR-146a and miR-124, to silence TNF-α gene expression in macrophages and T cells while concurrently inhibiting TNF-α-mediated NF-κB pathway activation, reducing TNF-α synthesis and release (128). Treg-EVs suppress TNF-α secretion in macrophages and Th17 cells by carrying anti-inflammatory cytokines, such as IL-10 and TGF-β (119, 120). Probiotic-derived EVs downregulate TNF-α expression in macrophages by blocking the TLR4/NF-κB signaling pathway via lipopolysaccharide derivatives (122).

As a key pro-inflammatory cytokine in RA, IL-6 promotes B-cell differentiation into plasma cells, activates Th17 cells, induces FLS proliferation and MMP secretion, and contributes to RA-associated complications such as anemia and osteoporosis (129, 130). EVs can modulate IL-6 secretion and signaling in RA through multiple mechanisms. MSC-EVs deliver miR-21 and miR-146a to target and silence IL-6 gene expression in macrophages and FLSs while inhibiting IL-6/JAK/STAT3 pathway activation (86). DC-EVs suppress IL-6 secretion in macrophages and T cells via delivery of IL-10 (121). SMSC-EVs downregulate IL-6 expression in FLSs through TGF-β and induce FLS apoptosis to reduce persistent cytokine secretion (131). Clinical studies report that IL-6 levels in peripheral blood EVs from RA patients are significantly elevated compared to healthy controls (*p* < 0.01) and positively correlate with DAS28 scores. Infusion of MSC-EVs significantly decreases peripheral blood IL-6 levels and improves DAS28, indicating that EVs can modulate RA disease activity by regulating the IL-6 signaling pathway (86).

IL-1β is a key initiator of synovial inflammation in RA, activating target cells, including macrophages and FLSs, which leads to excessive pro-inflammatory cytokine secretion and abnormal MMP expression, driving pathological joint tissue damage (132–134). EVs mediate anti-inflammatory effects by targeting IL-1β maturation and secretion. MSC-derived EVs exert dual regulatory effects. They deliver miR-146a and miR-223 to inhibit NLRP3 inflammasome activation in macrophages (117, 135). They transport antioxidant enzymes, including catalase and superoxide dismutase, to alleviate oxidative stress, which indirectly suppresses NLRP3 activation. These coordinated pathways result in decreased IL-1β release (136, 137). *In-vitro* studies further confirm that treatment of RA patient macrophages with MSC-EVs significantly downregulates NLRP3 and Caspase-1 mRNA expression and reduces IL-1β secretion, directly validating the targeted inhibitory effect of MSC-EVs on IL-1β (138).

IL-17, a central pro-inflammatory mediator in RA synovial inflammation and joint bone destruction, is primarily secreted by Th17 cells. It stimulates macrophages and FLSs to release pro-inflammatory cytokines and MMPs and recruits neutrophils to infiltrate synovial tissue. Its expression level positively correlates with the severity of RA-associated joint bone destruction (139, 140). EVs can suppress Th17-cell activation through multiple mechanisms, hence reducing IL-17 secretion. MSC-derived EVs deliver miRNAs such as miR-125b and miR-34a, which target and silence RORγt and STAT3 in Th17 cells, inhibiting their activation and proliferation (118). Treg-derived EVs directly inhibit Th17 activation by carrying IL-10 and TGF-β, while dendritic cell–derived EVs modulate the Th17/Treg balance via co-stimulatory molecules, including CD86 and CD40L. In CIA mouse models, infusion of MSC-EVs reduces the proportion of Th17 cells and IL-17 mRNA expression in synovial tissue, significantly alleviating joint bone destruction, demonstrating that EVs can mitigate RA joint damage by targeting IL-17 secretion (**Table 3**) (141).

**Table 3:** Summary of preclinical animal studies and clinical trials of EVs-based interventions for rheumatoid arthritis.

EVs formulation type	Study stage (preclinical/clinical)	Animal model/study population	Administration route	Primary outcome measures	Key study findings	References
Native MSC-EVs	Pre-clinical (animal study)	Collagen-induced arthritis (CIA) mouse model of RA	Tail vein injection/Intra-articular injection	Joint swelling degree, joint pathological score, DAS-equivalent score, serum RF/ACPA levels, pro/anti-inflammatory cytokines (TNF-α, IL-6, IL-10), synovial immune cell infiltration	①Significant relief of ankle joint swelling, reduced synovial hyperplasia and bone erosion in mice;② Markedly decreased serum RF, ACPA, IL-6, and TNF-α levels, upregulated IL-10 and TGF-β concentrations;③ Increased proportions of synovial M2 macrophages and Treg cells, reduced numbers of Th17 cells and plasma cells	(68, 114, 141)
IFN-β-pretreated MSC-EVs	*In-vitro* + preclinical	Primary peripheral blood lymphocytes from RA patients + CIA mice	*In-vitro* cell incubation, intravenous infusion in mice	Pro-inflammatory cytokine (IFN-γ, IL-2, TNF-α) secretion by CD4^+^ T cells, arthritis score in animals	Pretreated EVs exhibited superior inhibitory capacity on CD4^+^ T-cell pro-inflammatory cytokine production compared with native MSC-EVs, with enhanced *in-vivo* efficacy in suppressing pathogenic T-cell responses.	(102)
CTLA4Ig-engineered MSC-EVs (CT-EV)	Preclinical	CIA mice	Tail vein infusion	Th1/Th2/Th17/Treg subset ratios, joint pathological damage, serum cytokine levels	Significantly upregulated Th2 and Treg proportions and serum IL-4 levels, inhibited Th17 differentiation, suppressed synovial immune infiltration, and markedly reduced arthritis severity	(86)
Anti-CD80-Methotrexate conjugated engineered EVs	*In-vitro* cell study	Primary synovial macrophages from RA patients	*In-vitro* co-culture	Macrophage M1/M2 markers, pro-inflammatory cytokine secretion levels	Specifically targeted CD80^+^ pro-inflammatory macrophages, efficiently induced M1-to-M2 polarization, and drastically reduced local pro-inflammatory factor production	(75)
Native MSC-EVs	Preliminary clinical exploration (small-sample clinical trial)	Patients with active RA	Intravenous infusion	Peripheral blood IL-6 level, DAS28 disease activity score, clinical joint tenderness/swelling counts	Decreased peripheral blood IL-6 levels, significantly improved DAS28 scores, relieved clinical joint symptoms, and no serious adverse events were observed	(86)
Placental/embryo-derived circulating EVs (biomarker direction, non-therapeutic)	Clinical sample study	Normal pregnancy patients + patients with preeclampsia/pregnancy-associated arthritis	Peripheral blood sampling and detection	Plasma EVs-miRNA and EVs protein expression profiles	Altered EVs components can be used as non-invasive diagnostic biomarkers for pregnancy-associated autoimmune diseases for immune status monitoring	(41, 42)
Neutrophil-Derived EVs (Biomarker Study)	Clinical sample	Newly diagnosed RA patients vs. healthy controls	Synovial fluid and peripheral blood EVs isolation and sequencing	EVs particle size, concentration, miR-212-3p/miR-338-5p expression, neutrophil activation level	Differential expression of the above miRNAs can be used as novel liquid biopsy biomarkers for early screening and disease activity assessment of RA	(87, 88)

#### 3.3.2 Promotion of anti-inflammatory cytokines

Anti-inflammatory cytokines are central to maintaining immune homeostasis, suppressing pro-inflammatory factor secretion, regulating immune cell activation, and promoting tissue repair. In RA, the expression of these cytokines is significantly downregulated, limiting their capacity to counteract excessive inflammation. EVs modulate anti-inflammatory cytokine expression through multiple mechanisms, restoring immune balance and enhancing anti-inflammatory responses (142–144). Key anti-inflammatory cytokines in RA include IL-10, TGF-β, and IL-4, which EVs regulate with specificity and synergy. MSC-EVs carry IL-10 mRNA, miR-10a, and TGF-β-related molecules, promoting IL-10 and TGF-β secretion in target cells directly or indirectly. They also induce proliferation of macrophages, Tregs, and Bregs, amplifying anti-inflammatory effects (145). Studies demonstrate that MSC-EVs upregulate IL-10 mRNA in macrophages from RA patients and increase serum IL-10 and TGF-β concentrations in CIA mice. EVs further promote Th2 polarization by delivering miR-21, miR-125b, and CD40L, enhancing IL-4 secretion, restoring Th1/Th2 balance, and alleviating immune dysregulation (146). EVs also stimulate secretion of other anti-inflammatory factors, including IL-13 and IL-35, synergistically enhancing anti-inflammatory efficacy (147).

The core mechanisms by which EVs regulate anti-inflammatory cytokines can be summarized as follows: (1) delivery of specific molecules, such as miR-10a and miR-378a-5p, to directly modulate cytokine synthesis (72); (2) induction of M2 macrophage polarization, promoting anti-inflammatory factor secretion and forming positive feedback loops through engineered materials (148); (3) regulation of T-cell subset balance, restoring Treg function, and maintaining an immunosuppressive environment (103); (4) enhancement of anti-inflammatory efficacy via genetic modification, cell pretreatment, or physical engineering of EVs, such as CTLA4Ig overexpression, IFN-β pretreatment, and membrane elasticity engineering (85, 91, 102). In summary, EVs promote anti-inflammatory cytokine production and reshape the joint immune microenvironment in RA through multi-level mechanisms, including delivery of regulatory molecules, immune cell polarization, correction of T cell imbalance, and engineering modifications. These mechanisms provide a robust theoretical foundation and broad application potential for targeted RA therapy.

### 3.4 Roles of fibroblast-like synoviocyte- and platelet-derived extracellular vesicles in RA

Fibroblast-like synoviocyte- and platelet-derived extracellular vesicles act as pathogenic mediators driving systemic immune disturbance and focal joint damage in RA. By multi-dimensionally regulating inflammatory response, synovial hyperplasia, angiogenesis as well as bone and cartilage metabolism, these vesicles participate in the entire progression of pannus formation and irreversible structural joint destruction, serving as critical regulatory factors governing RA initiation and progression.

Extracellular vesicles derived from diseased fibroblast-like synoviocytes (FLS-EVs) function as core joint-specific pathogenic mediators within lesioned joints. Under the pathological microenvironment of RA, fibroblast-like synoviocytes predominantly secrete pro-pathogenic FLS-EVs to exacerbate local articular lesions. Loaded with pro-inflammatory cytokines including TNF-α, IL-6, and IL-8 as well as pro-inflammatory microRNAs such as miR-155 and miR-146a, FLS-EVs specifically target synovial macrophages and T lymphocytes. They trigger macrophage polarization toward the pro-inflammatory M1 phenotype and facilitate the differentiation of pro-inflammatory Th1/Th17 subsets, consequently remodeling and amplifying the intra-articular pro-inflammatory milieu persistently. Meanwhile, FLS-EVs recruit neutrophils to infiltrate synovial tissues, igniting cascading inflammatory reactions and sustaining chronic local joint inflammation (149–151). Via autocrine and paracrine signaling, FLS-EVs transport matrix metalloproteinases (MMP-1, MMP-3, and MMP-13) and ADAMTS proteases to disrupt the structural homeostasis of synovial stroma, provoke aberrant unlimited proliferation and invasive outgrowth of fibroblast-like synoviocytes into adjacent healthy connective tissues, and ultimately facilitate the formation of pathognomonic RA pannus (152, 153). Furthermore, FLS-EVs diffuse toward the surface of articular cartilage and osseous tissues, directly degrading core cartilaginous matrix constituents including type II collagen and proteoglycans. They also upregulate RANKL expression in bone marrow osteoclast precursors to accelerate osteoclast maturation and activation, enhancing bone resorption and triggering irreversible bone erosion (154).

Platelet-derived extracellular vesicles (Plt-EVs) represent pivotal circulating messengers bridging systemic immune dysregulation and localized joint injury in RA. Activated platelets abundantly release Plt-EVs into peripheral blood and synovial fluid of RA patients, and participate in RA pathogenesis through diverse pathways relying on the unique biological property of inflammation-coagulation crosstalk. On one hand, Plt-EVs carrying bioactive pro-inflammatory mediators (phosphatidylserine, IL-1β, TNF-α, leukotrienes) home preferentially to inflamed joints via systemic circulation, where they activate resident synovial fibroblasts and innate immune cells, linking peripheral autoimmune aberrance to localized synovitis and aggravating inflammatory severity (155). On the other hand, Plt-EVs loaded with master pro-angiogenic factors (VEGF, bFGF) robustly induce synovial angiogenesis to furnish sufficient oxygen and nutrients for pannus overgrowth and pathological synovial remodeling. Additionally, Plt-EVs elevate the systemic RANKL/OPG ratio in osteoblasts and osteoclast progenitors to promote osteoclastogenesis, while inducing chondrocyte apoptosis and cartilage matrix degradation, thereby exacerbating structural joint damage via dual impairment of bone turnover and cartilage integrity (156). Moreover, surface-enriched phosphatidylserine on Plt-EVs initiates the coagulation cascade to induce intra-synovial microthrombosis and microcirculatory stasis, resulting in accumulation of metabolic waste, aggravated chronic inflammatory infiltration and synovial fibrosis, which progressively fuels RA disease deterioration (156).

## 4 Clinical application status of extracellular vesicles in the treatment of RA

Benefiting from favorable biocompatibility and immunomodulatory properties, mesenchymal stem cell–derived extracellular vesicles (MSC-EVs) have emerged as a promising candidate for novel targeted therapy against rheumatoid arthritis (RA). Multiple early-phase clinical trials have preliminarily verified the safety and potential therapeutic efficacy of MSC-EVs in RA management. Intra-articular injection serves as the dominant administration route in ongoing clinical investigations. Available clinical data demonstrate satisfactory tolerability toward MSC-EVs among RA participants, with no severe adverse events reported throughout trials. Meanwhile, marked declines in Disease Activity Score 28 (DAS28) have been observed in enrolled patients, highlighting the therapeutic potential of MSC-EVs to mitigate inflammatory responses and retard disease progression of RA (157, 158). Small-scale open-label clinical studies have further uncovered the underlying functional mechanisms: MSC-EVs markedly reduce circulating pro-inflammatory cytokines in RA patients and elevate the proportion of peripheral regulatory T cells. By remodeling the dysregulated systemic immune milieu, MSC-EVs exert dual therapeutic effects including anti-inflammation and immune homeostasis restoration (159).

Nevertheless, current clinical investigations are subjected to prominent limitations. Most existing trials feature small sample sizes and non-controlled designs, compromising the reliability and generalizability of relevant research outcomes. Globally, standardized administration guidelines for MSC-EVs–based RA therapy remain absent, and clinical dosage is empirically determined referring to conventional mesenchymal stem cell regimens. Apart from local intra-articular injection, systemic intravenous administration is still in the exploratory infancy (160). According to available safety profiles, only mild adverse reactions such as injection-site pain, transient low-grade fever and slight hypersensitivity occur sporadically, whereas long-term risks associated with immunogenicity require continuous surveillance via long-term clinical follow-ups (161).

In summary, MSC-EVs possess distinct immunomodulatory merits and satisfactory biosafety, enabling promising prospects for clinical translation in RA treatment, as early clinical evidence has preliminarily validated their therapeutic effectiveness and manageable safety profile. However, several bottlenecks still hamper their clinical transformation, including imperfect trial design, lack of unified administration specifications and insufficient long-term safety evidence. In addition, technical obstacles consisting of inherent EV heterogeneity, unsatisfactory targeted delivery efficiency and challenges in standardized large-scale production substantially restrict the clinical translation of MSC-EVs.

## 5 EVs as targeted delivery vehicles

### 5.1 Natural targeting properties and modification strategies

EVs serve as key mediators of intercellular communication, with their inherent tissue- and cell-targeting capabilities constituting core advantages for drug delivery applications (162). This targeting is mediated by specific proteins, lipids, and glycosylation modifications on the EV membrane derived from the parent cell, which selectively bind to receptors on target cells, thus facilitating uptake (163). MSC-derived EVs, enriched with integrins and chemokine receptors, preferentially home to sites of inflammation or injury, such as inflamed joints in RA (164). Their high circulatory stability enables effective penetration of vascular endothelium and accumulation in synovial tissue, where they exert immunomodulatory and tissue-repairing effects (165). Therefore, native EVs, particularly MSC-EVs, show significant potential for treating inflammatory diseases (166). However, the targeting specificity, homing efficiency, and *in-vivo* half-life of native EVs are often insufficient to meet the precision requirements for complex diseases, including cancer and organ-specific injuries (167). To overcome these limitations, two major categories of surface engineering strategies have been developed, pre-separation and post-separation modifications, to achieve multifunctionalization and controlled drug release (168, 169). Post-isolation modifications, valued for flexibility and versatility, have been extensively studied and include chemical conjugation and click chemistry (170). For example, surface functionalization with monoclonal antibodies targeting epidermal growth factor receptors enables delivery systems that can cross the blood–brain barrier and transport Verrucarin A to glioblastoma (171).

Controlled drug release is a core focus in EV engineering. Techniques such as electroporation and pH-gradient-driven loading enable encapsulation of small molecules or nucleic acids within EVs or on their membranes (172, 173). Combining EVs with hydrogels creates localized sustained-release systems that extend drug action at target sites (174). The integration of these strategies positions engineered EVs as a smart nanoplatform for “targeting–drug loading–controlled release” applications (175). However, balancing modification efficiency with the preservation of intrinsic EV biological activity and establishing scalable, standardized production remain critical challenges for clinical translation (176).

### 5.2 Drug and gene delivery application

As natural nanocarriers, EVs show distinct advantages for drug and gene delivery in RA therapy. They efficiently encapsulate therapeutic molecules, including anti-inflammatory drugs, siRNA, and miRNA, and act by precisely regulating key pathological targets in RA (177). Platelet-derived EVs, exploiting their intrinsic inflammatory tissue tropism, accumulate in synovial tissue to deliver anti-inflammatory agents such as TPCA-1, mitigating local cytokine storms (178). In gene therapy, EVs loaded with CRISPR-Cas9 ribonucleoprotein complexes via electroporation or chemical transfection achieve targeted gene editing in Duchenne muscular dystrophy mouse models, demonstrating significantly higher delivery efficiency than free ribonucleoproteins (179, 180). Responsive release designs and engineering strategies further increase EV efficacy. ROS-sensitive hybrid EVs trigger drug release specifically at RA inflammation sites (181), while p^H^-responsive peptide-modified EVs improve intracellular delivery, achieving up to 73% siRNA-mediated gene silencing (182). Engineered EVs enable multifunctional and synergistic therapies. For instance, chondrocyte-targeted antibody-modified neutrophil EVs deliver combination therapies that selectively reduce synovial inflammation (183). MSC-EVs also remodel the joint immune microenvironment by delivering miR-214 (184). Despite these advances, challenges remain in standardizing drug-loading efficiency and in scaling production. Recent solutions involve 3D culture systems and hybrid EV technologies, with future integration of AI-assisted engineering approaches offering potential for personalized RA therapy (185).

### 5.3 Nanotechnology-enhanced EV therapies

The interdisciplinary integration of nanotechnology and EVs offers novel approaches to treating chronic inflammatory diseases, such as RA, by significantly improving targeting, safety, and therapeutic efficacy. A key innovation involves hybridization of nanomaterials with EVs, leveraging complementary advantages through biomimetic masking, fusion, or nanostructure-mediated regulation. Fusion of EVs with synthetic nanoparticles, such as gold nanoparticles or liposomes, generates hybrid systems that retain EVs’ intrinsic targeting capabilities while incorporating nanoparticles’ functional properties. For example, fusion of M1 macrophage-derived EVs with thermoresponsive liposomes enabled dual functions of tissue targeting and temperature-dependent drug release (186). Cholesterol-modified multivalent DNA structures (PX-DNA-chol), constructed via DNA nanotechnology, act as “synthetic nano-glue” to rapidly aggregate EVs into micron-scale clusters, facilitating enrichment and efficient separation through low-speed centrifugation (187). Surface functionalization with ligands, such as CD44 aptamers, further enhances EV targeting of synovial fibroblasts (188).

Physically stimulated delivery strategies provide an additional layer of control, using ultrasound or magnetic fields to modulate EV biodistribution and release. Ultrasonic cavitation increases EV penetration and retention in inflamed joints while triggering controlled drug release. For instance, EVs loaded with zinc oxide nanocrystals (ZnO NCs) (TrojanNanoHorse, TNH) showed increased lymphoma cytotoxicity upon shock wave activation and demonstrated increased anti-inflammatory effects in RA models (189). Magnetic field-guided delivery systems employing superparamagnetic iron oxide nanoparticles (SPIONs) achieve precise EV targeting to joint sites alongside controlled release (190). Clinical translation of these hybrid strategies remains limited by challenges, including standardizing large-scale production, optimizing drug-loading efficiency, and conducting long-term safety evaluations. Integration of microfluidic technologies with AI-assisted design has the potential to advance RA treatment (191). Future research should focus on optimizing nanomaterial–EV combinations and developing multimodal, stimulus-responsive delivery platforms.

## 6 Challenges and limitations of EV-based RA therapies

### 6.1 Standardization, preparation, and quality control

The clinical translation of EVs as therapeutic vehicles depends on the establishment of standardized preparation and quality control systems. Core challenges involve precise regulation of purity, yield, and batch-to-batch consistency. Studies indicate that even when identical separation methods, such as TFF, are used, EVs from different sources (e.g., MSC-EVs) may be insufficiently pure due to co-isolated proteins, such as albumin (192). This directly affects functional evaluation and therapeutic efficacy, highlighting the need for rigorous quality control.

The MISEV2018 guidelines from the ISEV provide a framework for EV characterization, including marker detection (e.g., CD63, TSG101) and double-lipid-layer visualization via TEM. However, methodological variations remain across laboratories; for instance, equine bone marrow MSC-derived EVs show inconsistent characterization outcomes (193), indicating that standardized practices are not yet widely implemented.

Technical bottlenecks are evident in three areas. (1) Purification efficiency: ultracentrifugation with density gradients (UC-DG) reduces lipoprotein contamination (194), while SEC may retain non-vesicular particles (195). (2) Accurate quantification: NTA is affected by parameter variability (196), whereas quartz crystal microbalance (QCM) can differentiate vesicles from non-vesicular particles (197). (3) Functional validation: standardized methodologies for assessing EV functionality remain insufficient. Emerging technologies, including detectEV platforms and microfluidics combined with machine learning, offer promising solutions for functional quality assessment (198, 199). Addressing these challenges requires multidisciplinary approaches: scalable EV production via automated bioreactors, comprehensive characterization using omics techniques, the establishment of reference materials, and the refinement of ISEV guidelines, integrated with intelligent platforms. These strategies are essential for advancing EVs from experimental research to clinical application.

### 6.2 *In-vivo* kinetics and safety concerns

For the clinical translation of EVs as therapeutic carriers or drug-delivery systems, their *in-vivo* kinetics and long-term safety pose critical bottlenecks that must be addressed. Current evidence indicates that the *in-vivo* distribution, metabolic patterns, and potential immunogenicity of EVs remain incompletely characterized. Studies employing radionuclide or fluorescent labeling have elucidated aspects of EV fate: following intravenous administration, human umbilical cord MSC-EVs (UCMSC-EVs) are predominantly sequestered by the liver and spleen, displaying a rapid distribution phase and a relatively short elimination phase in blood half-life (200); similarly, zirconium-89-labeled hUCMSC-EVs show high uptake in liver and spleen and persistent accumulation at tumor sites in tumor-bearing mice (201), indicating significant reticuloendothelial system (RES) capture after systemic EV delivery, which may compromise targeting efficiency to inflamed joints. Moreover, the metabolic pathways of EVs remain poorly defined; orally administered small EVs (sEVs) can resist gastrointestinal degradation and reach systemic circulation via intestinal absorption (202). As allogeneic biomaterials, EVs demonstrate low immunogenicity (203); however, surface antigen variability among EVs from different cell or donor sources may elicit immune responses. Systematic evaluation of batch consistency, long-term *in-vivo* distribution, clearance mechanisms, and immune effects is necessary to ensure safety in chronic disease applications (204). Furthermore, EVs’ immune-evasion capacity and nonspecific effects limit the precision of therapy. Unmodified EVs display nonspecific biodistribution and potential organ toxicity, whereas intrinsic bioactive molecules may induce unpredictable effects. Surface engineering modifications, combined with rigorous production quality control, are essential for reducing heterogeneity, therefore ensuring therapeutic specificity and safety (205).

### 6.3 Clinical translation and regulatory oversight

As an emerging therapeutic approach for RA, the clinical translation of EVs encounters three primary bottlenecks: dose standardization, optimization of administration routes, and establishment of efficacy evaluation systems. Current clinical trials indicate that the dose-response relationship of EVs remains undefined, with significant variability in *in-vivo* distribution and half-life among EVs derived from distinct sources, including MSCs and immune cells (206). Drawing on experience from lipid nanovesicle dose optimization in cancer immunotherapy, individualized administration protocols should consider EV particle size, surface protein composition, and drug-loading characteristics (207). Regarding delivery strategies, intra-articular injection permits localized high-concentration administration, whereas systemic modulation of inflammation necessitates combined intravenous and subcutaneous routes. Development of RA-specific targeting modification technologies is required to improve bioavailability (208). Efficacy assessment should extend beyond traditional DAS28 scoring to include a comprehensive evaluation of EV-mediated regulation of Th17/Treg balance and dynamic alterations in synovial fluid inflammatory cytokine profiles (209). The regulatory landscape for EVs remains exploratory, with no global consensus on classification: the US FDA categorizes EVs as biological products under 21 CFR Part 600, whereas the EU EMA applies Advanced Therapy Medicinal Product (ATMP) regulations (210). Core regulatory priorities include production quality control (e.g., ISO 13485 certification), process stability (e.g., comparison of ultracentrifugation *and SEC), and safety assessment (e.g., tumorigenicity and* immunogenicity) (211). Veterinary studies highlight that interspecies applications require stringent validation of EV heterogeneity and host compatibility (212). To facilitate translation, the ISEV MISEV2023 guidelines advocate for standardized characterization protocols (e.g., FCM for CD9/CD63/CD81) and integration of AI-assisted quality control (213). Policy frameworks should balance innovation with risk by expediting approval for refractory RA indications via “breakthrough therapy” designations, while ensuring long-term follow-up to monitor potential adverse events (214).

## 7 Conclusion and future perspectives

Research on EVs in RA therapy has advanced from fundamental mechanistic studies to a focus on clinical translation, establishing EVs as a prominent target in autoimmune disease treatment. Current evidence demonstrates that EVs, particularly MSC-EVs, provide innovative therapeutic strategies for RA that exceed the capabilities of conventional drugs and cell therapies, owing to their low immunogenicity, biocompatibility, and multi-targeted immunomodulatory functions (215). Based on biogenesis and morphological size, EVs are classified into subcategories, including exosomes, microvesicles, and apoptotic bodies. By transporting bioactive molecules, such as miRNAs and proteins, they replicate parent-cell functions to regulate macrophage polarization and T/B-cell balance, systematically remodeling the disrupted joint immune microenvironment. As natural nanoscale delivery vehicles, EVs establish a foundation for precision RA therapy (216). In RA immune modulation, EVs emulate parent-cell functions by regulating innate and adaptive immune cells. They restore M1/M2 macrophage polarization equilibrium, inhibit neutrophil activation and NET formation, modulate dendritic cell maturation, rebalance Th17/Treg subsets, suppress aberrant B-cell activation, and reduce autoantibody secretion (e.g., RF, ACPA). Furthermore, EVs bidirectionally regulate the RA cytokine network, suppressing pro-inflammatory cytokines such as TNF-α, IL-6, IL-1β, and IL-17 and promoting anti-inflammatory cytokines, including IL-10 and TGF-β. This coordinated regulation remodels the joint immune microenvironment, with MSC-EVs alleviating inflammation by delivering miR-146a, which inhibits the NF-κB pathway. Regarding targeted delivery, EVs exploit intrinsic targeting properties to efficiently transport therapeutic molecules, including anti-inflammatory drugs, siRNA, and miRNA. Integration of surface engineering and nanotechnology enables the construction of multifunctional “targeting-drug loading-controlled release” platforms, enhancing both precision and therapeutic efficacy in RA. These developments provide a robust foundation for advancing precision RA therapy.

EVs possess promising clinical application prospects in immune regulation and targeted therapy for RA. However, current studies still face multiple academic controversies, experimental system defects, and clinical transformation barriers, which greatly restrict the high-quality development and clinical implementation of EV-based RA therapies. EVs exhibit distinct dual pro-inflammatory and anti-inflammatory regulatory effects during RA pathogenesis. Their biological functions are not static but are highly dependent on parental cell sources, inflammatory microenvironments, vesicle subtypes, and working concentrations, which fundamentally explain the inconsistent conclusions among existing studies. Specifically, EVs derived from healthy stem cells and resting immune cells can effectively restore immune homeostasis, inhibit inflammatory responses, and alleviate joint damage. In contrast, EVs secreted by activated synovial cells and immune cells in the inflammatory microenvironment of RA patients undergo pathological molecular remodeling, which activates inflammatory signaling pathways such as NF-κB, amplifies inflammatory cascades, and accelerates disease progression. There exists essential functional heterogeneity between EVs derived from healthy individuals and RA patients. Furthermore, the biological activities of EVs vary significantly between active and remission stages of RA. Current studies predominantly focus on the anti-inflammatory and therapeutic potential of healthy cell-derived EVs while neglecting the pathogenic mechanisms of patient-derived EVs, leading to one-sided understanding in this research field. At present, preclinical RA studies heavily rely on collagen-induced arthritis (CIA) mouse models. As an acute and self-limiting inflammatory model, CIA cannot fully recapitulate the chronic, recurrent, and systemic pathological characteristics of human RA, including persistent synovial hyperplasia and immune memory formation, resulting in poor clinical translation of preclinical findings. In addition, unified standardized research systems for EVs have not been established. The inconsistency in EV isolation and purification techniques, identification criteria, experimental parameters, and functional evaluation indicators remains prevalent. Conventional methods such as ultracentrifugation suffer from low yield, insufficient purity, and poor batch stability, causing poor experimental repeatability and prominent research heterogeneity. Collectively, the clinical transformation of EV-based RA therapy encounters multiple core bottlenecks. The precise molecular mechanisms underlying the dual immune regulatory effects of EVs remain unclear; GMP-grade standardized preparation and quality control systems as well as mature large-scale production technologies are lacking; the *in-vivo* kinetic distribution and long-term biosafety of EVs have not been fully clarified; and high-quality, large-sample, multicenter randomized controlled trials (RCTs) are absent. Moreover, standardized administration regimens and unified efficacy evaluation systems have not been established, and relevant industry regulatory specifications remain incomplete, which severely hinders the clinical transformation and popularization of EV therapies for RA.

Future research on RA EV therapy should follow a coordinated pathway of “mechanism deepening–technology optimization–clinical validation,” progressing synergistically along three principal directions. Multidisciplinary collaboration across materials science, nanotechnology, and clinical medicine will facilitate translation from fundamental research to clinical application. At the mechanistic level, investigations should focus on EV-mediated cellular communication networks and immune regulatory pathways. Detailed analysis is needed to elucidate the interactions between EVs and specific immune cells, such as Th17/Treg subsets, using techniques such as single-cell sequencing to delineate their spatiotemporal distribution within the RA microenvironment (216). Furthermore, characterization of novel EV subpopulations, including migratory EVs, may provide insights into cell migration and immune evasion and represent critical targets for future mechanistic breakthroughs.

At the level of technological translation and industrialization, the development of efficient and controllable engineered production methods is essential to overcome current technical bottlenecks (217). Priorities include microfluidic high-throughput separation, genetic engineering of donor cells, and bionic synthesis of EVs (218). Strategies to extend intra-articular retention, such as freeze-dried formulations or hydrogel-based sustained-release systems (219), are important to integrate the complex synergistic effects of native EVs with the precise targeting of engineered EVs. This approach enables minimally modified, maximally effective therapeutic designs that synergize with conventional DMARDs or biologics.

At the industrial and clinical level, establishing GMP-grade production standards, exploring cost-effective alternative sources such as milk-derived EVs, and systematically evaluating the impact of standardized storage on EV functionality are essential (220). Multicenter RCTs should support clinical translation by determining dose-response relationships, optimal administration routes, and long-term safety, and by using larger sample sizes to validate efficacy. Unified quality control standards based on ISEV guidelines are necessary to mitigate functional variability arising from EV heterogeneity. Integration with dynamic biomarker monitoring, such as citrullinated proteins, will facilitate personalized therapy. Adopting a phased clinical translation strategy of “local injection first, gradual expansion” will support the development of next-generation “smart” combination therapy systems for RA, offering safer, more effective, and durable treatment options while advancing progress in the management of autoimmune diseases.
